# Has multimorbidity and frailty in adult hospital admissions changed over the last 15 years? A retrospective study of 107 million admissions in England

**DOI:** 10.1186/s12916-024-03572-z

**Published:** 2024-09-11

**Authors:** Puji Faitna, Alex Bottle, Bob Klaber, Paul P. Aylin

**Affiliations:** 1 School of Public Health, Imperial College London, 80-92 Wood Lane, London, W12 7TA UK; 2 Imperial College London Healthcare NHS Trust, St Mary’s Hospital, South Wharf Road, London, W2 1NY UK

**Keywords:** Adult, Frailty, Frail, Older people, Multimorbidity, Comorbidity, Hospitalisation, Ageing, Hospitals, Socioeconomic Factors, Inpatients

## Abstract

**Background:**

Few studies have quantified multimorbidity and frailty trends within hospital settings, with even fewer reporting how much is attributable to the ageing population and individual patient factors. Studies to date have tended to focus on people over 65, rarely capturing older people or stratifying findings by planned and unplanned activity. As the UK’s national health service (NHS) backlog worsens, and debates about productivity dominate, it is essential to understand these hospital trends so health services can meet them.

**Methods:**

Hospital Episode Statistics inpatient admission records were extracted for adults between 2006 and 2021. Multimorbidity and frailty was measured using Elixhauser Comorbidity Index and Soong Frailty Scores. Yearly proportions of people with Elixhauser conditions (0, 1, 2, 3 +) or frailty syndromes (0, 1, 2 +) were reported, and the prevalence between 2006 and 2021 compared. Logistic regression models measured how much patient factors impacted the likelihood of having three or more Elixhauser conditions or two or more frailty syndromes. Results were stratified by age groups (18–44, 45–64 and 65 +) and admission type (emergency or elective).

**Results:**

The study included 107 million adult inpatient hospital episodes. Overall, the proportion of admissions with one or more Elixhauser conditions rose for acute and elective admissions, with the trend becoming more prominent as age increased. This was most striking among acute admissions for people aged 65 and over, who saw a 35.2% absolute increase in the proportion of admissions who had three or more Elixhauser conditions. This means there were 915,221 extra hospital episodes in the last 12 months of the study, by people who had at least three Elixhauser conditions compared with 15 years ago. The findings were similar for people who had one or more frailty syndromes. Overall, year, age and socioeconomic deprivation were found to be strongly and positively associated with having three or more Elixhauser conditions or two or more frailty syndromes, with socioeconomic deprivation showing a strong dose–response relationship.

**Conclusions:**

Overall, the proportion of hospital admissions with multiple conditions or frailty syndromes has risen over the last 15 years. This matches smaller-scale and anecdotal reports from hospitals and can inform how hospitals are reimbursed.

**Supplementary Information:**

The online version contains supplementary material available at 10.1186/s12916-024-03572-z.

## Background

As in many Westernised countries, in England, the health service faces big challenges due to an ageing population, with estimates reporting that two-thirds of people over 65 have at least two long-term health conditions [1]. Yet, little is known about how people over 65 utilise healthcare services, despite having the worst health outcomes [1]. Evidence has tended to focus on individual health conditions, and there have been recent calls to move towards a more systematic approach that acknowledges the realities of patients with multiple health conditions [2]. This presents significant challenges to the NHS [3, 4].

Multimorbidity can be defined as a condition characterised by the coexistence of multiple chronic conditions (two or more), producing a complex clinical presentation that is more than just the sum of the diseases coexisting in an individual [1]. Multimorbidity can strongly impact the quality of life, mental health [5], daily function and healthcare use of individuals [1, 6]. People with multiple conditions can have significant direct—through increased primary and secondary care use and prescribing—and indirect costs—through loss of work productivity and increased absenteeism, which tend to affect individuals who have not retired. Age is an important driver of multimorbidity [7], particularly within high-income countries [8]. The epidemiology of multimorbidity is driven by many factors, including socioeconomic deprivation [8, 9], the health condition itself and ‘lifestyle choices’ such as poor diet and lack of exercise [10].

There are many ways to measure and define multimorbidity and frailty. Comorbidity is a related but distinct concept and can be defined as a health condition in addition to an index health condition by an individual [1]. Frailty can be defined as a set of clinically recognised syndromes occurring more among vulnerable older persons [11]. The concept of frailty and its definition has evolved over time, and we elaborate on this in more detail in the discussion.

Multimorbidity and frailty are associated with increased mortality, falls, disability and hospitalisations and measuring its prevalence is important because they impact health service utilisation and health care delivery. Hospital care for older people living with frailty can be expensive and lengthy, and building a stronger evidence base for evidence-based clinical practice guidelines to improve the safety and quality of care is a key research priority [12]. The costs of frailty alone on health service utilisation has been estimated to be £6 billion per year to the NHS across primary and secondary care [13]. Frailty is an emerging public health priority with major implications for clinical practice and public health, and its prevalence is expected to rise [14].

Despite people who are over 65 with multiple conditions having comparatively worse health outcomes, and people who are under 65 comprising the largest age group with multiple conditions in absolute terms, little is known about how they utilise healthcare, let alone at a national level [10, 12, 15]. More large-scale cohort studies on frailty are needed to support the evidence underpinning clinical practice guidelines and closing any gaps between evidence and practice [12], especially given that older people are often omitted from clinical trials [16]. This study utilises multimorbidity as an overarching concept encompassing conditions and frailty and uses national hospital administrative data to measure time trends.

Hypothesis: Multimorbidity in adult hospital admissions has increased over the last 15 years.

Primary aim: To describe how the proportion of admissions by people who are multimorbid has changed over time.

Secondary aims: To test and quantify how much ageing impacts multimorbidity trends and if patient characteristics or interactions impact the trend.

## Methods

### Data sources and cohort definition

England’s Hospital Episode Statistics (HES) admitted patient care data is an administrative dataset provided by NHS Digital which captures all hospital episodes except for private patients treated in privately run hospitals. HES classifies diseases using the 10th standardised International Classification of Diseases (ICD-10) coding system. Adult episodes between March 2006 and March 2021 were extracted and results grouped into people aged between 18 and 44 years (18–44 s), 45 and 64 years (45–64 s) and people aged 65 years and over (65 +). A wide age range was included because older people and younger individuals are not often captured in national cohort studies [7]. Results were stratified into elective and acute admissions to reflect the clinically distinct populations and act as a proxy for what is modifiable by the hospital (e.g. elective admissions). HES captures all episodes by NHS patients treated within any NHS hospital or private hospital. A spell is typically composed of multiple linked consultant episodes, and where a spell ended in a transfer to another NHS hospital, it was linked to become a ‘superspell’, which allowed each unit of measurement to be an ‘admission’. Transfers were captured on an admission level, so multiple transfers within a single admission were counted as a single transfer. Medical and surgical specialties were derived from previously published work [17]. The study end date of March 2021 was selected based on the data available during the analysis. Area-level socioeconomic deprivation in HES was measured using the Index of Multiple Deprivation, which comprises seven domains, such as income and unemployment [18]. Emergency admissions refer to any unplanned inpatient admissions, usually via an emergency department visit.

### Multimorbidity and frailty

Long-term conditions were identified from the primary or secondary diagnosis codes using the International Statistical Classification of Diseases, Injuries and Causes of Death (ICD-10) codes. The number of conditions was defined using the Elixhauser comorbidity index [19, 20]. Dementia was added to the conditions score and is based on the validated Charlson Index [21], as many comorbidity measures tend to omit markers of cognitive frailty, which may underestimate multimorbidity prevalence [22]. The conditions included were chronic heart failure, arrhythmia, valvular disease, diseases of the pulmonary circulation, peripheral vascular disease, hypertension, paralysis, other neurological diseases, chronic pulmonary diseases, diabetes, thyroid disorders, renal disease, liver disease, peptic ulcers, cancer, connective tissue disorders, coagulopathy, obesity, weight loss, fluid disorders, anaemia, alcohol-related diseases, substance abuse, psychoses, depression and dementia. Composite measures were generated to reflect distinct conditions rather than severity for the following: hypertension (combined hypertension with complications and hypertension without complications), diabetes (combined diabetes with complications and diabetes without complications), cancer (combined lymphoma, metastases and solid tumour) and anaemia (combined blood loss anaemia and deficiency anaemia).

The frailty score was based on Soong et al.’s Frailty Score, which uses ICD-10 diagnostic codes within all diagnostic fields [11, 23]. The included frailty syndromes were as follows: dementia, delirium, mobility problems, falls and fractures, pressure ulcers, incontinence, dependence and care, anxiety and depression and senility. If an individual had two frailty syndromes coded within the same admission, they would have been coded as having two of the frailty syndromes.

### Statistical analyses

Annual trends were reported over 15 years, with each admission falling within four possible comorbid condition categories (0, 1, 2 or 3 +) or three frailty syndrome categories (0,1 or 2 +). Logistic regression model outcomes were admissions of people with at least three conditions and at least two frailty syndromes, respectively. The prevalence for the first and last year of the study were compared and reflect admissions from April 2006 to March 2007 and April 2020 to March 2021, respectively.

Annual admission proportions were plotted on the *y*-axis, while the *x*-axis reflected calendar year. Admissions by people who are over 110 years comprised a very small proportion (< 0.001%) and were winsorised as we assumed that these admissions were most likely a data entry error.

Logistic regression models quantified how much of the trend was attributable to age, and their slopes compared for people over 65. The base models (models 1, 3, 5, 7) contained only year as a covariate, while the comparative models (models 2, 4, 6, 8) also included age as a continuous variable. Age was plotted to assess linearity (Additional File 1: Fig. S1-S4), and spline knots were fitted at 90 and 103 for conditions and 100 for frailty, to account for non-linearity.

To quantify how much year was attenuated by patient factors or interactions between (1) age and year, (2) gender and year and (3) socioeconomic deprivation and year, base models (multimorbidity: M9, 13; frailty: M17, 21) were compared with each interaction term separately (multimorbidity: M10–12, 14–16; frailty: M18–20; 22–24). For simplicity and ease of interpretation, age was categorised.

### Sensitivity analysis

To test if an increased trend reflected improved coding quality, rather than a true rise, a sensitivity analysis was run that reviewed the quality of the coding for the diagnosis fields before, during and after mandatory changes were made to the ICD-10 comorbidity coding guidelines, irrespective of speciality [24]. In 2011, with the overarching aim to improve the clinical coding of comorbidities, there was an update in the guidance for how comorbidity within HES inpatient admissions were recorded and coded for [24]. From 2011 to 2013, a list of conditions was released, which meant that clinical coders were mandated to code these conditions when reviewing patients’ medical records [24–26]. We hypothesised that if the clinical coding was in fact poor, and that the rise in multimorbidity and frailty was because of improvements in clinical coding, we would expect to see a stepwise change in the proportion of total admissions coded with diabetes, depression or dementia when the mandated coding was introduced. We extended the sensitivity analysis by age group (18 to 44 years, 45 to 64 years and 65 years and older) and by acute and elective admission, to further test if improvements in the clinical coding of multimorbidity and frailty differed by age group or admission type. We selected diabetes, depression and dementia for the sensitivity analysis as they are either conditions within the Elixhauser Comorbidity Index and Soong’s Frailty Score or were part of the first release stage of conditions [26]. The plots conservatively represented coding trends between 2009 and 2015, as the earliest documentation found was from 2010.

## Results

Table 1 reported patient characteristics admissions by multimorbidity and frailty count and the study included 106 million inpatient hospital admissions.

**Table 1:** Patient characteristics

Feature	Value	Multimorbidity				Frailty syndromes		
		**0**	**1**	**2**	**3 + **	**0**	**1**	**2 + **
	*N* (%)	42 M 39.9	26 M 24.7	18 M 16.6	20 M 18.9	85 M 79.9	17 M 15.9	4.5 M 4.2
Age (years)	Mean (SD^a^) 18–44 45–64 65 +	41.4 (18.9) 66.7 18.2 15.2	56.5 (20.8) 31.6 28.1 40.3	65.7 (18.4) 15.0 25.9 59.1	72.5 (15.1) 5.8 19.7 74.6	52.1 (21.5) 42.5 23.0 34.5	63.3 (22.1) 24.0 21.6 54.4	79.3 (14.4) 4.0 8.9 87.0
Gender	Male Female	28.6 71.4	43.0 57.0	47.6 52.5	49.5 50.5	38.9 61.1	41.3 58.7	38.1 62.0
Socioeconomic Deprivation Quintile	Quintile 1 (Least deprived) Quintile 2 Quintile 3 Quintile 4 Quintile 5 (Most deprived) Quintile 6 (Unknown)	16.7 18.0 19.3 20.9 24.2 1.0	17.4 19.3 19.9 20.6 21.9 1.0	16.7 19.3 20.2 21.0 22.1 0.8	15.5 18.8 20.4 21.6 23.2 0.6	16.7 18.6 19.6 20.8 23.3 0.9	15.9 18.6 20.2 21.7 22.7 0.9	17.2 20.0 21.3 21.1 20.0 0.5
Ethnic group	Black or Black British Asian or Asian British White Other (incl. mixed) Unknown	3.7 7.1 73.8 3.9 11.6	2.3 4.2 80.2 2.3 11.1	1.9 3.9 83.6 1.8 8.7	2.1 4.2 85.6 1.5 6.6	3.1 6.0 77.8 3.0 10.3	1.5 2.8 84.4 1.8 9.5	1.3 2.0 87.9 1.3 7.5
Admission source	Home Transfer Other/unknown	96.3 2.9 0.7	95.4 3.6 1.0	95.0 4.0 1.0	94.2 4.5 1.3	96.1 3.2 0.8	93.7 4.9 1.4	91.4 5.8 2.8
Emergency admissions in previous 12 months	0 1 2 3 +	48.1 30.4 21.8 16.1	24.9 25.2 24.0 22.8	14.3 19.6 21.4 22.1	12.7 24.8 32.8 39.0	67.5 17.6 6.9 8.0	55.5 20.6 9.9 14.0	43.4 25.2 13.8 17.7
Admission type	Emergency Elective	47.3 52.7	66.2 33.9	75.7 24.3	83.5 16.5	57.4 42.7	85.6 14.4	96.8 3.2
Specialty	Medical Surgical	70.7 29.3	69.2 30.8	71.9 28.1	78.8 21.2	70.9 29.1	74.8 25.2	83.2 16.9

^a^Standard deviation

### Multimorbidity trends

**Fig. 1:**
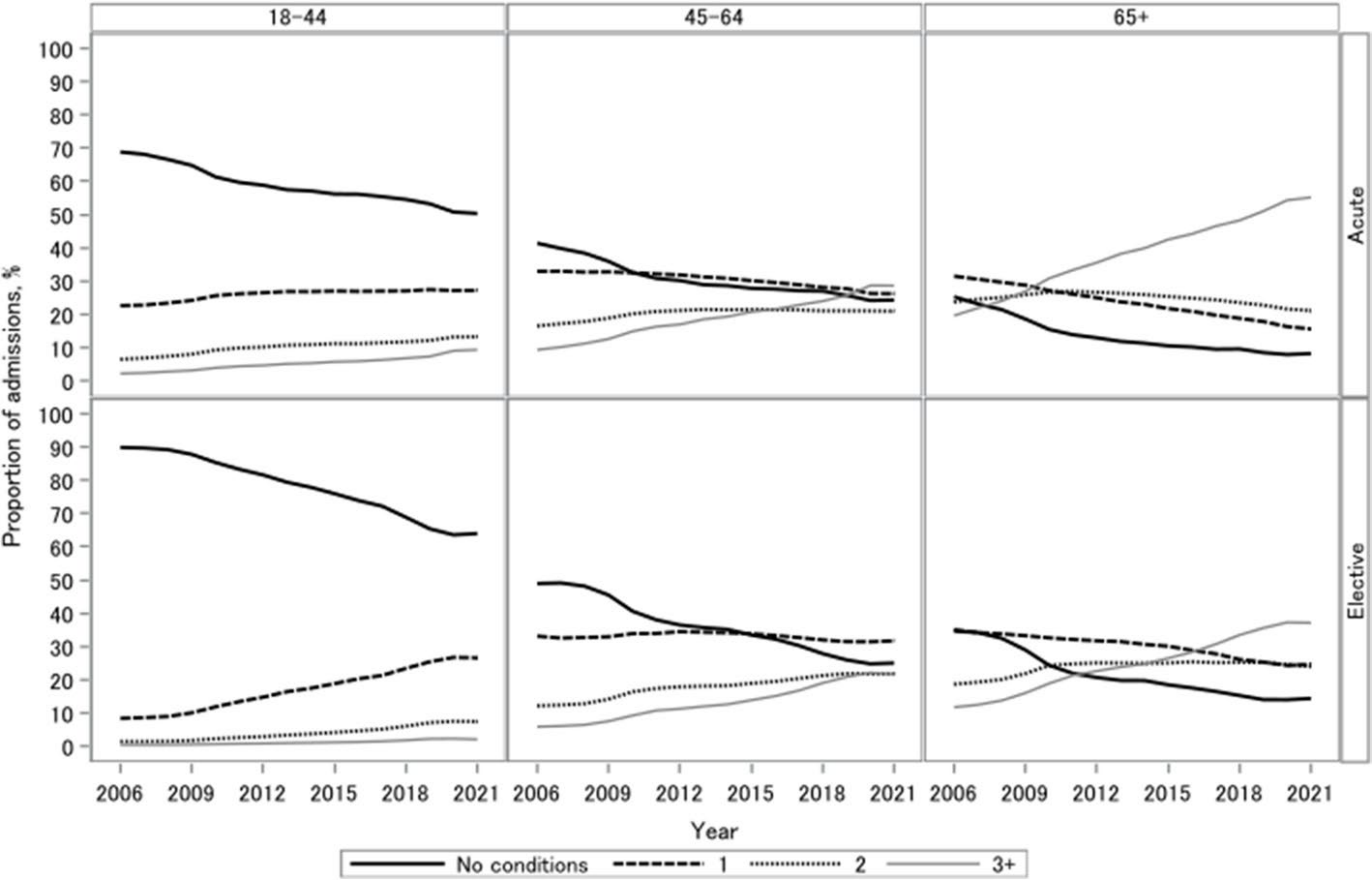
Proportion of admission trends with 0 to 3 + conditions by age and admission type

Overall, when comparing the prevalence between 2006 with 2021, the proportion of admissions with at least one Elixhauser condition rose for elective and acute admissions, with the trend becoming more prominent as age increased (Fig. 1). The acute admissions gradient was more striking among people with three or more Elixhauser conditions than elective admissions. The prevalence for all Elixhauser conditions increased over time when we compared the first and last year of the study. The prevalence of depression and obesity saw a stark rise, increasing from 1.9 to 10.1% and 0.7 to 9.2%, respectively (Additional File 1: Table S1).

#### Comorbidity trends for acute admissions

This proportional rise over the last 15 years was most striking among people who had three or more Elixhauser conditions, with absolute proportions rising by 35.2% (2006: 19.8%; 2021: 55.0%), 19.7% (2006: 9.4%; 2021: 29.1%) and 7.3% (2006: 2.2%; 2021: 9.5%) for people who were over 65, the 45–64 s and 18–44 s, respectively (Additional File 1: Table S2). This meant there were 915,221, 249,460 and 81,819 extra hospital admissions by people who had three or more Elixhauser conditions in 2021 compared with 2006 for people aged over 65, 45–64 and 18–44, respectively. There was a 4.6% (2006: 22.6%; 2021: 27.1%) and 7.0% (2006: 6.5%; 2021: 13.4%) increase in the proportion of admissions with one or two Elixhauser conditions among the 18–44 s (Additional File 1: Table S2). For the 45–64 s, there was a 7.0% reduction (2006: 33.0%; 2021: 25.9%) in people with one Elixhauser condition and a 4.5% increase in people with two Elixhauser conditions (2006: 16.6%; 2021: 21.0%) (Additional File 1, Table S2).

Conversely, although the proportion of admissions who had no Elixhauser conditions was higher among those under 65, the drop over time was relatively uniform (18–44: − 18.8% (2006: 68.8%; 2021: 50.0%); 45–64: − 17.2% (2006: 41.1%; 2021: 24.0%); 65 + : − 17.2% (2006: 25.0%; 2021: 7.8%)) (Additional File 1: Table S2). In absolute terms, the largest increase in admissions was among people over 65 with three or more Elixhauser conditions, as they had 915,221 extra admissions in 2021 compared with 2006.

#### Comorbidity trends for elective admissions

A similar trend was observed for elective admissions, where there was a higher proportion of admissions with Elixhauser conditions among the acute compared with elective admissions. For people aged 45 years and over, the proportion of admissions with at least two or more Elixhauser conditions increased, with the increase being most prominent among the people with at least three Elixhauser conditions (65 + : 25.6% (2006: 11.8%; 2021: 37.5%); 45–64 s: 16.2% (2006: 5.9%; 2021: 22.2%)), followed by people with two Elixhauser conditions (65 + : 5.5% (2006: 18.7%; 2021: 24.2%); 45–64 s: 9.4% (2006: 12.2%; 2021: 21.6%)) (Additional File: Table S2). This equates to an additional 48,892 hospital admissions by people over the age of 65 with at least three Elixhauser conditions in 2021 compared with 2006.

Overall, the largest absolute increase in elective admissions was by people aged between 18 and 44 with at least one Elixhauser condition, as they had 165,377 extra admissions in 2021 compared with 2006. For people aged 18 to 44, the largest proportional increase over the last 15 years was among people with one Elixhauser condition, increasing by 18.3% (2006: 8.4%; 2021: 26.7%) (Additional File 1: Table S2). The proportion of admissions by people with at least two Elixhauser conditions increased over time but to a lesser extent, with a 6.1% (2006: 1.4%; 2021: 7.5%) increase and 1.7% (2006: 0.4%; 2021: 2.2%) increase among people with at least three Elixhauser conditions (Additional File 1: Table S2).

### Frailty trends

**Fig. 2:**
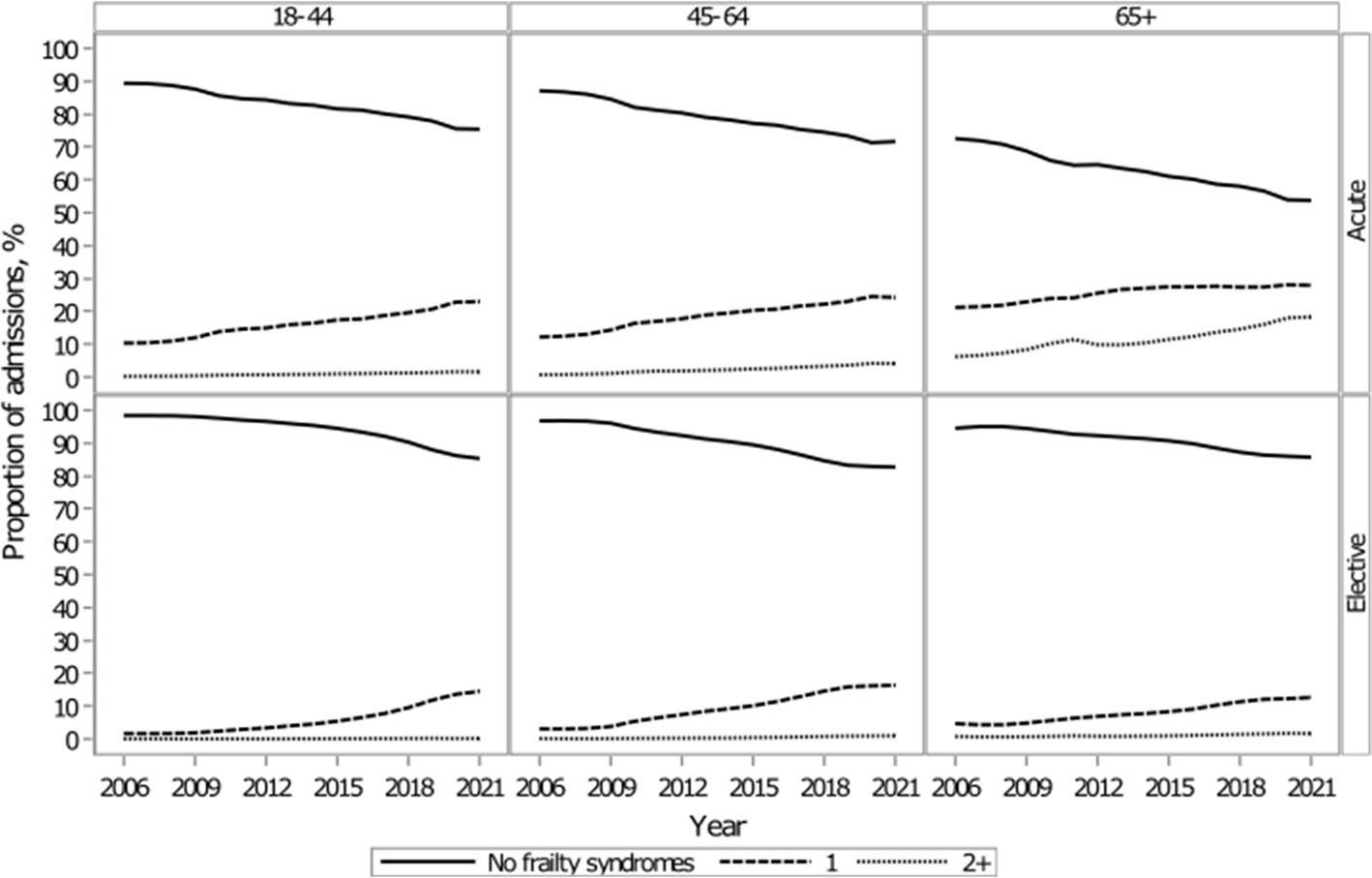
Proportion of admission trends with 0 to 2 + frailty syndromes by age and admission type

Overall, when observing the proportion of admissions with at least one frailty syndrome, there was an increase across acute and elective admissions, and, like with multimorbidity, this trend was more evident with increasing age (Fig. 2). The prevalence for each frailty syndrome increased with exception to ‘dependence and care’ when we compared the first year with the last year of the study. The prevalence of ‘dementia’ and ‘anxiety and depression’ rose notably over time, increasing from 2.9 to 6.8% and from 2.2 to 14.3%, respectively (Additional File 1: Table S3).

#### Frailty trends for acute admissions

For acute admissions, there was a higher proportion of admissions by people with at least one frailty syndrome in 2021 than compared with 2006. There was a similar rise in frailty syndromes across age groups, but the rise was most apparent among people with one frailty syndrome, with a 13.2% (2006: 10.3%; 2021: 23.4%) increase by people aged 18 to 44 and a 12.6% increase by people aged between 45 and 64 (2006: 12.1%; 2021: 24.8%) (Additional File 1:Table S4). There were 177,399 and 148,406 extra admissions by people with one frailty syndrome in 2020/2021 compared with 15 years ago. For people aged over 65, there was a 12.2% (2006: 6.3%; 2021: 18.4%) increase among people with two or more frailty syndromes (Additional File 1: Table S4). This meant 312,569 extra admissions by people over 65 who had at least two frailty syndromes in 2020/2021, compared with 15 years ago.

Among those aged 18–44 and 45–64, the proportion of admissions with at least two frailty syndromes increased, but to a lesser extent than for people with only one frailty syndrome (18–44 s: 1.4% (2006: 0.3%; 2021: 1.7%); 45–64 s: 3.6% (2006: 0.7%; 2021: 4.3%)) (Additional File 1: Table S4). For people over 65, the proportion of admissions with one frailty syndrome increased by 7.1% (2006: 21.1%; 2021: 28.2%) (Additional File 1: Table S4). In absolute terms, the largest increase in admissions was among people over 65 with at least two frailty syndromes, with an additional 312,569 admissions in 2020/2021 compared with 15 years ago.

#### Frailty trends for elective admissions

For elective admissions, there was an increase in admissions where patients were frail, which was mostly made up of people with one frailty syndrome. There was a 12.4% (2006: 1.6%; 2021: 14.0%) and 13.1% (2006: 3.1%; 2021: 16.1%) increase among people aged 18–44 s and 45–64 s, respectively (Additional File 1: Table S4). People aged 65 and over with one frailty syndrome also saw a 7.8% (2006: 4.6%; 2021: 12.4%) increase (Additional File 1: Table S4). There were proportional increases among people who had at least frailty syndromes, but the proportions were much smaller and increased by approximately 1% (18–44: 0.2% (2006: 0.03%; 2021: 0.2%); 45–64: 0.8% (2006: 0.2%; 2021: 1.0%); 65 + : 1.0% (2006: 0.8%; 2021: 1.8%)) (Additional File 1: Table S4). In absolute terms, the largest increase in admissions over the last 15 years was among people aged 18 to 44 with one frailty syndrome, with an extra 127,381 admissions in 2021.

### Admission trends attributable to age

Table 2 showed that for people 65 and over, adjusting for age did not have a big impact on the effect size for year, and that age had a non-linear relationship on comorbidity and frailty.

**Table 2:** Odds ratios quantifying condition and frailty trends attributable to age among people 65 and over

Outcome	Admission type	Model number	Covariates	Odds ratio (OR) (95% CI)	*p*-value
Condition	Acute	1 (base)	Year	1.11 (1.11 to 1.11)	< 0.0001
		2	Year	1.11 (1.11 to 1.11)	< 0.0001
			Age (years):		
			65–90	1.03 (1.03 to 1.03)	< 0.0001
			90–103	0.94 (0.94 to 0.94)	< 0.0001
			> 103	0.70 (0.69 to 0.71)	< 0.0001
	Elective	3 (base)	Year	1.11 (1.11 to 1.11)	< 0.0001
		4	Year Age:	1.11 (1.11 to 1.11)	< 0.0001
			65–90	1.03 (1.03 to 1.03)	< 0.0001
			90–103	0.92 (0.91 to 0.92)	< 0.0001
			> 103	0.50 (0.44 to 0.97)	< 0.0001
Frailty	Acute	5 (base)	Year	1.08 (1.08 to 1.08)	< 0.0001
		6	Year Age:	1.08 (1.08 to 1.08)	< 0.0001
			65–100	1.08 (1.08 to 1.08)	< 0.0001
			> 100	0.78 (0.70 to 0.78)	< 0.0001
	Elective	7 (base)	Year	1.07 (1.07 to 1.07)	< 0.0001
		8	Year Age:	1.07 (1.07 to 1.07)	< 0.0001
			65–100	1.10 (1.10 to 1.11)	< 0.0001
			> 100	0.70 (0.70 to 0.74)	< 0.0001

#### Comorbidity

After accounting for age for people over 65, with each passing year, the odds of having three or more Elixhauser conditions increased by 11% for both admission types. The odds of someone having two or more frailty syndromes increased by 7 to 8% (Table 2).

The odds for people aged between 65 to 90 years, with three or more Elixhauser conditions, increased by 3% for each 1-year increase in age for both admission types, after accounting for calendar year (Table 2). Among people aged between 90 and 103, the odds of having three or more Elixhauser conditions decreased by 6 to 8% and then dropped even further by 30 to 50% when people were over 103 years old.

#### Frailty

A similar trend was observed for frailty across both admission types. The odds of a person with at least two frailty syndromes increased by 7 to 8% for each calendar year from the age of 65. For admissions by people between 65 and 100, every 1-year increase in age was associated with an 8 to 10% increase having two or more frailty syndromes. Beyond 100 years of age, the odds decreased by 22% and 30% for acute and elective and admissions, respectively.

### Interactions for comorbidity

Interactions between year and patient factors (age, gender and socioeconomic deprivation) had little impact on attenuating the relationship between year and the likelihood of having three or more Elixhauser conditions (Table 3). For both admission types, the interaction terms were statistically significant, but the effect size was small, representing a minor clinical effect. For instance, had the interaction term effect size for model 15 (which tested the interaction between year and gender) been larger, this would indicate that the rate of having at least three comorbidities grew much faster among females than in males.

**Table 3:** Comparing the odds ratios for having at least three Elixhauser conditions and testing for interactions, stratified by admission type

Conditions					
		**Acute**		**Elective**	
Covariates	Value	OR (95% CI)	*p*	OR (95% CI)	*p*
Base models		Model 9		Model 13	
Year		1.11 (1.11 to 1.11)	< 0.0001	1.12 (1.12 to 1.12)	< 0.0001
Age	18–44 45–64 65 +	1 4.42 (4.41 to 4.43) 12.96 (12.93 to 12.99)	- < 0.0001 < 0.0001	1 13.44 (13.38 to 13.51) 30.82 (30.68 to 30.96)	- < 0.0001 < 0.0001
Gender	Male Female	1 0.90 (0.90 to 0.90)	- < 0.0001	1 0.77 (0.77 to 0.77)	- < 0.0001
Socioeconomic deprivation Quintile	Quintile 1 (Least deprived) Quintile 2 Quintile 3 Quintile 4 Quintile 5 (Most deprived) Quintile 6 (Unknown)	1 1.14 (1.13 to 1.14) 1.29 (1.29 to 1.29) 1.48 (1.48 to 1.48) 1.71 (1.71 to 1.72) 1.09 (1.08 to 1.09)	- < 0.0001 < 0.0001 < 0.0001 < 0.0001 < 0.0001	1 1.16 (1.15 to 1.16) 1.32 (1.32 to 1.33) 1.57 (1.56 to 1.57) 1.86 (1.85 to 1.86) 1.59 (1.57 to 1.62)	- < 0.0001 < 0.0001 < 0.0001 < 0.0001 < 0.0001
Interaction 1		Model 10		Model 14	
Year		1.10 (1.10 to 1.10)		1.14 (1.14 to 1.14)	< 0.0001
Age	18–44 45–64 65 +	1 4.60 (4.57 to 4.62) 11.36 (11.30 to 11.41)	-	1 16.54 (16.32 to 16.73) 38.43 (38.02 to 38.86)	- < 0.0001 < 0.0001
Gender	Male Female	1 0.90 (0.90 to 0.90)	-	1 0.77 (0.77 to 0.77)	- < 0.0001
Socioeconomic Deprivation Quintile	Quintile 1 (Least deprived) Quintile 2 Quintile 3 Quintile 4 Quintile 5 (Most deprived) Quintile 6 (Unknown)	1 1.14 (1.13 to 1.14) 1.29 (1.29 to 1.29) 1.48 (1.48 to 1.49) 1.72 (1.72 to 1.72) 1.09 (1.08 to 1.10)	- < 0.0001 < 0.0001 < 0.0001 < 0.0001 < 0.0001	1 1.16 (1.15 to 1.16) 1.32 (1.32 to 1.33) 1.56 (1.56 to 1.57) 1.85 (1.85 to 1.86) 1.59 (1.56 to 1.61)	- < 0.0001 < 0.0001 < 0.0001 < 0.0001 < 0.0001
Interaction between Year and Age	Year*18–44 Year*45–64 Year*65 +	1 1.00 (0.99 to 1.00) 1.02 (1.02 to 1.02)	- < 0.0001 < 0.0001	1 0.98 (0.98 to 0.98) 0.98 (0.97 to 0.98)	- < 0.0001 < 0.0001
Interaction 2		Model 11		Model 15	
Year		1.11 (1.11 to 1.11)	< 0.0001	1.11 (1.11 to 1.11)	< 0.0001
Age	18–44 45–64 65 +	1 4.42 (4.41 to 4.43) 12.97 (12.94 to 12.99)	- < 0.0001 < 0.0001	1 13.48 (13.41 to 13.54) 30.90 (30.75 to 31.04)	- < 0.0001 < 0.0001
Gender	Male Female	1 0.88 (0.87 to 0.88)	- < 0.0001	1 0.73 (0.73 to 0.73)	- < 0.0001
Socioeconomic deprivation	Quintile 1 (Least deprived) Quintile 2 Quintile 3 Quintile 4 Quintile 5 (Most deprived) Quintile 6 (Unknown)	1 1.14 (1.13 to 1.14) 1.29 (1.29 to 1.29) 1.48 (1.48 to 1.48) 1.71 (1.71 to 1.72) 1.09 (1.08 to 1.09)	- < 0.0001 < 0.0001 < 0.0001 < 0.0001 < 0.0001	1 1.16 (1.15 to 1.16) 1.32 (1.32 to 1.33) 1.57 (1.56 to 1.57) 1.86 (1.85 to 1.86) 1.59 (1.57 to 1.62)	- < 0.0001 < 0.0001 < 0.0001 < 0.0001 < 0.0001
Interaction between Year and Gender	Male Female	1 1.00 (1.00 to 1.00)	- < 0.0001	1 1.01 (1.01 to 1.01)	- < 0.0001
Interaction 3		Model 12		Model 16	
Year		1.10 (1.10 to 1.10)	< 0.0001	1.11 (1.11 to 1.11)	
Age	18–44 45–64 65 +	1 4.42 (4.41 to 4.43) 12.99 (12.96 to 13.02)	- < 0.0001 < 0.0001	1 13.46 (13.39 to 13.52) 30.88 (30.73 to 31.02)	- < 0.0001 < 0.0001
Gender	Male Female	1 0.90 (0.90 to 0.90)	- < 0.0001	1 0.77 (0.77 to 0.77)	- < 0.0001
Socioeconomic Deprivation Quintile	Quintile 1 (Least deprived) Quintile 2 Quintile 3 Quintile 4 Quintile 5 (Most deprived) Quintile 6 (Unknown)	1 1.09 (1.08 to 1.09) 1.19 (1.19 to 1.20) 1.36 (1.35 to 1.37) 1.55 (1.54 to 1.56) 0.94 (0.92 to 0.95)	- < 0.0001 < 0.0001 < 0.0001 < 0.0001 < 0.0001	1 1.11 (1.10 to 1.12) 1.24 (1.23 to 1.25) 1.47 (1.45 to 1.48) 1.74 (1.72 to 1.75) 1.38 (1.34 to 1.43)	- < 0.0001 < 0.0001 < 0.0001 < 0.0001 < 0.0001
Interaction between Year and Socioeconomic Deprivation Quintile	YearQuntile 1 (Least deprived) Year*Quntile 2 Year*Quntile 3 Year*Quintile 4 Year*Quintile 5 (Most deprived) Year*Quintile 6 (Unknown)	1 1.01 (1.00 to 1.01) 1.01 (1.01 to 1.01) 1.01 (1.01 to 1.01) 1.01 (1.01 to 1.01) 1.02 (1.02 to 1.02)	- < 0.0001 < 0.0001 < 0.0001 < 0.0001 < 0.0001	1 1.01 (1.01 to 1.01) 1.01 (1.01 to 1.01) 1.01 (1.01 to 1.01) 1.01 (1.01 to 1.01) 1.02 (1.01 to 1.02)	- < 0.0001 < 0.0001 < 0.0001 < 0.0001 < 0.0001

* = interaction between

### Interactions for frailty

Interactions between year and patient factors (age, gender and socioeconomic deprivation) had mixed results on how this attenuated the relationship between calendar year and the odds of having two or more frailty syndromes (Table 4). Like with comorbidity, across both admission types, the interaction terms were statistically significant but had small effect sizes, representing a minor clinical effect. The main exception to this is model 22, which tested the interaction between year and age among elective admissions. The rate of having at least two frailty syndromes was 8% (*p* < 0.0001) lower among people who are 65 and over than when compared with people aged 18 to 44.

**Table 4:** Comparing the OR for two or more frailty syndromes and testing for interactions by admission type

Frailty syndromes					
		**Acute**		**Elective**	
Covariates	Value	OR (95% CI)	*p*	OR (95% CI)	*p*
Base models		Model 17		Model 21	
Year		1.09 (1.08 to 1.09)	< 0.0001	1.11 (1.10 to 1.11)	< 0.0001
Age	18–44 45–64 65 +	1 2.77 (2.75 to 2.79) 15.22 (15.14 to 15.30)	- < 0.0001 < 0.0001	1 4.54 (4.45 to 4.62) 12.12 (11.93 to 12.32)	- < 0.0001 < 0.0001
Gender	Male Female	1 1.51 (1.51 to 1.52)	- < 0.0001	1 1.20 (1.19 to 1.21)	- < 0.0001
Socioeconomic Deprivation Quintile	Quintile 1 (Least deprived) Quintile 2 Quintile 3 Quintile 4 Quintile 5 (Most deprived) Quintile 6 Unknown)	1 1.03 (1.03 to 1.04) 1.10 (1.10 to 1.11) 1.14 (1.14 to 1.14) 1.14 (1.13 to 1.14) 1.07 (1.05 to 1.08)	- < 0.0001 < 0.0001 < 0.0001 < 0.0001 < 0.0001	1 1.13 (1.11 to 1.14) 1.26 (1.24 to 1.28) 1.42 (1.39 to 1.44) 1.53 (1.50 to 1.56) 1.58 (1.48 to 1.68)	- < 0.0001 < 0.0001 < 0.0001 < 0.0001 < 0.0001
Interaction 1		Model 18		Model 22	
Year		1.12 (1.12 to 1.12)	< 0.0001	1.17 (1.17 to 1.18)	< 0.0001
Age	18–44 45–64 65 +	1 2.79 (2.75 to 2.83) 21.07 (20.81 to 21.34)	- < 0.0001 < 0.0001	1 4.55 (4.34 to 4.76) 26.16 (25.16 to 27.19)	- < 0.0001 < 0.0001
Gender	Male Female	1 1.51 (1.51 to 1.51)	- < 0.0001	1 1.19 (1.18 to 1.21)	- < 0.0001
Socioeconomic Deprivation Quintile	Quintile 1 (Least deprived) Quintile 2 Quintile 3 Quintile 4 Quintile 5 (Most deprived) Quintile 6 (Unknown)	1 1.03 (1.03 to 1.03) 1.10 (1.10 to 1.11) 1.14 (1.14 to 1.14) 1.13 (1.13 to 1.14) 1.06 (1.05 to 1.08)	- < 0.0001 < 0.0001 < 0.0001 < 0.0001 < 0.0001	1 1.12 (1.10 to 1.14) 1.25 (1.23 to 1.27) 1.40 (1.37 to 1.42) 1.50 (1.48 to 1.53) 1.54 (1.44 to 1.64)	- < 0.0001 < 0.0001 < 0.0001 < 0.0001 < 0.0001
Interaction between Year and Age	Year*18–44 Year*45–64 Year*65 +	1 1.00 (1.00 to 1.00) 0.97 (0.96 to 0.97)	- 0.0330 < 0.0001	1 1.00 (1.00 to 1.01) 0.92 (0.91 to 0.92)	- 0.5221 < 0.0001
Interaction 2		Model 19		Model 23	
Year		1.09 (1.09 to 1.09)	< 0.0001	1.10 (1.10 to 1.10)	< 0.0001
Age	18–44 45–64 65 +	1 2.77 (2.75 to 2.79) 15.20 (15.12 to 15.28)	- < 0.0001 < 0.0001	1 4.54 (4.46 to 4.62) 12.14 (11.94 to 12.33)	- < 0.0001 < 0.0001
Gender	Male Female	1 1.71 (1.70 to 1.72)	- < 0.0001	1 1.14 (1.11 to 1.16)	- < 0.0001
Socioeconomic Deprivation Quintile	Quintile 1 (Least deprived) Quintile 2 Quintile 3 Quintile 4 Quintile 5 (Most deprived) Quintile 6 (Unknown)	1 1.03 (1.03 to 1.04) 1.10 (1.10 to 1.11) 1.14 (1.14 to 1.14) 1.14 (1.13 to 1.14) 1.06 (1.05 to 1.08)	- < 0.0001 < 0.0001 < 0.0001 < 0.0001 < 0.0001	1 1.13 (1.11 to 1.14) 1.26 (1.24 to 1.28) 1.42 (1.39 to 1.44) 1.53 (1.50 to 1.56) 1.58 (1.48 to 1.68)	- < 0.0001 < 0.0001 < 0.0001 < 0.0001 < 0.0001
Interaction between Year and Gender	Male Female	1 0.99 (0.99 to 0.99)	- < 0.0001	1 1.01 (1.00 to 1.01)	- < 0.0001
Interaction 3		Model 20		Model 24	
Year		1.08 (1.08 to 1.08)	< 0.0001	1.09 (1.09 to 1.09)	< 0.0001
Age	18–44 45–64 65 +	1 2.77 (2.75 to 2.79) 15.24 (15.16 to 15.32)	- < 0.0001 < 0.0001	1 4.54 (4.46 to 4.62) 12.16 (11.97 to 12.36)	- < 0.0001 < 0.0001
Gender	Male Female	1 1.51 (1.51 to 1.52)	- < 0.0001	1 1.20 (1.19 to 1.21)	- < 0.0001
Socioeconomic Deprivation Quintile	Quintile 1 (Least deprived) Quintile 2 Quintile 3 Quintile 4 Quintile 5 (Most deprived) Quintile 6 (Unknown)	1 1.04 (1.03 to 1.04) 1.06 (1.05 to 1.07) 1.07 (1.06 to 1.08) 1.02 (1.01 to 1.03) 1.01 (0.98 to 1.04)	- < 0.0001 < 0.0001 < 0.0001 < 0.0001 0.5988	1 1.08 (1.04 to 1.21) 1.08 (1.03 to 1.12) 1.17 (1.13 to 1.22) 1.19 (1.14 to 1.23) 1.69 (1.48 to 1.93)	- 0.0258 < 0.0001 < 0.0001 < 0.0001 0.2198
Interaction between Year and Socioeconomic Deprivation Quintile	Year*Quintile 1 (Least deprived) Year*Quintile 2 Year*Quintile 3 Year*Quintile 4 Year*Quintile 5 (Most deprived) Year*Quintile 6 (Unknown)	1 1.00 (1.00 to 1.00) 1.00 (1.00 to 1.00) 1.01 (1.01 to 1.01) 1.01 (1.01 to 1.01) 1.01 (1.00 to 1.01)	- 0.2265 < 0.0001 < 0.0001 < 0.0001 0.0002	1 1.01 (1.00 to 1.01) 1.02 (1.01 to 1.02) 1.01 (1.01 to 1.03) 1.03 (1.03 to 1.03) 0.99 (0.98 to 1.01)	- 0.0258 < 0.0001 < 0.0001 < 0.0001 0.2198

### Sensitivity analysis

The sensitivity analysis showed no clear step change in clinical coding was observed for all admissions for people aged 18–44 (Additional File 1: Fig. S5), 45 to 64 (Additional File 1: Fig. S6) or 65 and over (Additional File 1: Fig. S7) for dementia, depression and diabetes. Similarly, no clear step change in clinical coding was observed for the aforementioned conditions when stratified by elective (Additional File 1: Fig. S8) and emergency (Additional File 1: Fig. S9) inpatient admissions.

## Discussion

### Main findings

These results are the first nationwide study of adult comorbidity and frailty prevalence in hospitals from 2006 to 2021. Overall, the proportion of admissions where adults had an Elixhauser conditions or frailty syndrome increased over time, and this became more prominent as age increased. This was most striking among acute admissions by people over 65 with at least three Elixhauser conditions, which increased by 35.2% over the last 15 years, equating to an extra 915,221 hospital admissions. Age had a non-linear relationship with the likelihood of being a person with multimorbidity among people over 65. For every 1-year increase in age, the odds of having three or more Elixhauser conditions increased by 3% and around 10% for people with at least two frailty syndromes. These odds then began to drop as patients became nonagenarians and centenarians. Overall, the tested interactions were significant but had a minor clinical effect.

### Comparison with previous literature

Our findings are broadly consistent with the multimorbidity and frailty literature in secondary care acute settings [27–30], adding to studies focused on specialised cohorts like emergency general surgery [28], peri-operative [29] and older populations [28–30]. A 2023 international meta-analysis [27] of 45 observational studies on emergency admissions estimated moderate to severe frailty prevalence ranged from 16.1 to 66.9%. Similarly, we found the proportion of admissions with at least one frailty syndrome ranged from 18.4 to 28.2% among people aged over 65. Similarly, a frailty study on acute general surgery patients who were over 65 across five sites [28] found 74.0% had at least two conditions, while we found a 76.0% prevalence in the final year of our study. In contrast, the same study found 28.0% of their cohort were ‘mildly frail or above’, while we reported 46.6% had at least one frailty syndrome in 2021. Soong et al. analysed a cohort of people aged over 65 between 2005 and 2013, using the same dataset, and found frailty coding had increased from 12% to 14% and that anxiety and/or depression had increased from 2.4% to over 4.0% [30]. For the same age group, but using more recent data, we found the presence of frailty had increased from 6.3 to 18.4%, and a similar but more rapid rise in anxiety and/or depression (2.2 to 14.3%). A UK study focused on perioperative patients [29] found their patients were, on average, 2.3 years older and more complex in 2021 than when compared with 2013, which supports our findings that increasing age is significantly associated with increased conditions over time.

### Defining frailty

Early definitions of frailty described this condition as a phenotype that included exhaustion, loss of physical performance, increased dependence in daily activities and involuntary weight loss [31]. Then, the deficit accumulation model of frailty [32] was introduced, which aimed to more explicitly recognise the complexity of frailty, defining it as when an individual has a large number of co-occurring identifiable medical conditions, which include functional deficits and comorbidities. More specialised fields, like geriatrics and gerontology, have defined frailty as a syndrome characterised by a clinically recognisable state of increased vulnerability, resulting from an ageing-associated decline in reserve and function across multiple physiological systems, such that the ability to cope with the everyday or acute stressors is compromised. There is a need to quantify frailty, especially when using administrative hospital datasets; however, there are limitations in how well they can capture more granular details like functional deficits or subjective symptoms. Thus, there is a need to use proxy measures instead. Soong’s Frailty Score [23] is one way to operationalise frailty syndromes, as it is a proxy measure for frailty using administrative hospital data. Soong et al. defined frailty as ‘a combination of the pathophysiological consequence of inbuilt senescence and the accumulation of defects throughout a life course…[which] appears to be related to, but distinct from, disability, comorbidity and chronological age’ [23, 33].

### Possible drivers of the trend in comorbidity and frailty

In primary care, multimorbidity has been strongly associated with ageing and socioeconomic deprivation [7]. People who are more socioeconomically deprived are more likely to have multiple conditions, which can occur more than a decade earlier than when compared with the wealthy. This has been reported in multiple studies, across primary [7, 8, 34, 35] and secondary [36, 37] healthcare settings. One argument is that multimorbidity is socially patterned and that healthcare and society-related developments are possible explanations for this observed trend [6, 28]. A 2023 report by England’s Chief Medical Officer further supports this, positing older age and poverty as the two greatest drivers of multimorbidity in England [38]. Female gender has also been widely associated with frailty [15, 37], with a recent international systematic review of cohort studies reporting frailty prevalence as higher among females [14], which could reflect coding improvements or the higher proportion of women who tend to live to an older age compared with men [39]. The rising trend in multimorbidity and frailty could also be influenced by increased hospital readmission rates by complex patients, but further studies would be needed to test this.

### Changes in the population over time

One possible interpretation of the rise in multimorbidity is that it serves as a proxy of the population becoming less healthy over time. This interpretation cannot be answered using our study as our focus was on hospitalised patients, who would fundamentally be more unwell compared with the general population. Health Survey for England reports are better suited to answer this as they report on population health trends and health-related behaviours that match the start and end points of our study. Health Survey for England reports a mixed picture, with doctor-diagnosed diabetes [33, 34], obesity [33, 34] and extreme anxiety or depression [40] worsening over time and cardiovascular and respiratory health [40] and smoking [41] improving over time.

### Non-linear relationship between age and comorbidity and frailty

Splines were administered to statistically account for the drop observed between age and multimorbidity once patients became nonagenarians and centenarians. The drop was unexpected but is consistent with findings from another study using administrative health data that found that the likelihood of having more conditions flattened as patients approached very advanced age and attributed some of this to a ‘selectivity effect’ [42], whereby the multimorbid tended not to reach 100.

Alternatively, one study [43] that reviewed post-mortems of centenarians posited that centenarians may be an intrinsically healthier birth cohort who consequently experience disease onset later in life because of beneficial adaptations and genetics. Our findings could also further their argument that because the cause of death among centenarians is often due to organ failure rather than advanced age or senile debility, which challenges the notion that advanced age is a disease. Similarly, the 2023 Chief Medical Officer’s Annual Report on Health in an Ageing Society [38] acknowledged that although some, particularly people who are frail, will need significant care and support as they reach older age, the majority of people in the UK who are in their late 80s and 90s do not live in care homes [44]. This challenges the common assumption that older age is automatically associated with significant increases in care and support needs. Further studies would need to be undertaken to replicate these findings in a larger and more diverse cohort before being able to generalise these findings to the whole population.

Other explanations for the non-linear relationship between age and comorbidity and frailty include possible selection bias among surgeons performing elective operations, whereby elective surgeries are only performed on the healthiest older people. People in their 90s and onwards could also be more likely to present unconscious or unable to provide their own histories, impacting their ability to report their histories, or their clinical histories comprehensively could be so extensive that, rather than filling out the entire history, clinicians may use previous admissions instead. Lastly, people in their 90s and onwards might also prefer being treated at home or within community settings rather than be hospitalised, possibly skewing our selection of the older people.

### Policy implications

Older people with multiple conditions can significantly impact healthcare services [45] by increasing demands on peri-operative pre-assessments, increasing complexities during surgery, having longer anaesthetic and surgery times and increasing the need for limited critical care beds [29]. These trends can also impact waiting lists and operating theatre capacity, and some have called for an upscaling of current services [29].

Hospital payment systems need to take multimorbidity into account and reward accordingly, as it is more complex to manage people with multiple conditions as they are likely to stay in hospital longer, and have more complex discharge processes. This has an impact on the number of cases a service or hospital are able see, impacting on its productivity. From a clinical perspective, knowing which associations are most common helps to tailor care and identify appropriate therapeutic approaches and interventions. From a policy perspective, a better understanding of factors related to multimorbidity can help policies move toward preventative approaches that focus on delaying or slowing down its progression.

### Strengths and limitations

A key strength of this study is that it used two much-used measures which allowed for robust findings. The metrics have previously been used on administrative whole population data [46], strengthening the reproducibility and comparability across different datasets. Second, including people who are under 65 and in their 70s and beyond meant that an often understudied age group, due to a lack of data or very small sample sizes, could be analysed. Third, stratifying trends across acute and elective admissions allowed for a more nuanced understanding of which pathways were most affected. Fourth, 15 years provided a robust and broad perspective of time trends. Fifth, this study benefited from a large national cohort with a very large dataset that is suitable for informing health service planning and where primary diagnosis and procedure fields is > 95% accurate, with some secondary fields subject to some under recording [47].

Limitations included variations in diagnostic coding practices for comorbidities between hospitals [18], which we attempted to address by comparing our findings with the Health Survey of England. Second, the steady rise could be due to staggered improvements in clinical coding by hospitals or the introduction of electronic patient records, which we tried to mitigate through our sensitivity analysis and found no clear stepwise change when mandatory coding was introduced. Third, there were large variations in how multimorbidity [35] is measured, and we addressed this by using commonly used coding systems and metrics. Fourth, there are ongoing debates on how conditions are grouped and how accurately they reflect complexity. We tried to address this by grouping condition subcategories into a single condition so that each condition in the metric reflected a distinct condition rather than a mixture of conditions and severity [46]. Fifth, analysing administrative data meant that we are unable to capture clinical information such as medications and daily support for living. Sixth, the last year of our study was during the COVID-19 pandemic, so caution is needed when making comparisons between the start and end of the study, as we cannot be certain about how the pandemic may have biased the epidemiology of hospital admissions for this cohort. However, the observed trends in multimorbidity and frailty before the pandemic showed a steady increase over the study period. Seventh, Soong’s Frailty Score has typically been applied to the older adult population, but to our knowledge, there was no evidence to suggest that these findings would not be accurate in the younger adult population. Lastly, grouping people over 65 in the analysis may not fully capture the trends of different decades. However, we did this to enable a simplified interpretation of coefficients.

### Future research directions

Our study focused on the broad trends in hospital admissions for adult individuals with multimorbidity or frailty syndromes over the last 15 years. Further research could build upon our findings and test whether these national trends in multimorbidity and frailty were associated with prolonged hospitalisation, complications or hospital readmissions. Similarly, future studies could measure the financial implications of these increased trends on hospitals. Furthermore, studies could investigate trends among the more clinically complex individuals who have four or more long-term conditions or quantify how much of the trend is driven by possible increased readmissions by more clinically complex patients. Future studies could also investigate if the observed hospital admission trends in multimorbidity and frailty, and their respective mix of long-term conditions or syndromes, vary by gender or geographical region.

## Conclusions

Overall, the proportion of admissions into hospital by adults with multiple conditions or frailty syndromes has risen in the last 15 years. It is important we understand how these trends have impacted hospital services, and the wider systems of care, to improve the quality and safety of healthcare services and inform their future planning.

## Supplementary Information

Additional File 1: Fig. S1: Age vs log (odds) of three or more comorbidities for acute admissions across England from 2006 to 2021. Fig. S2: Age vs log (odds) of three or more comorbidities for elective admissions across England from 2006 to 2021. Fig. S3: Age vs log (odds) of two or more frailty syndromes for acute admissions across England from 2006 to 2021. Fig. S4: Age vs log (odds) of two or more frailty syndromes for elective admissions across England from 2006 to 2021. Table S1: List of conditions used to measure multimorbidity and their point prevalence at the beginning and end of the study. Table S2: Percentages for the proportion of admission trends with 0 to 3+ conditions by age and admission type for 2006/7 and 2020/2021. Table S3: List of syndromes used to measure frailty and their point prevalence at the beginning and end of the study. Table S4: Percentages for the proportion of admission trends with 0 to 2+ frailty syndromes by age and admission type for 2006/7 and 2020/2021. Fig. S5: Proportion of all inpatient HES admissions with dementia, depression or diabetes aged 18-44 years from 2009 to 2015. Fig. S6: Proportion of all inpatient HES admissions with dementia, depression, or diabetes aged 45-64 years from 2009 to 2015. Fig. S7: Proportion of all inpatient HES admissions with dementia, depression, or diabetes aged 65+ from 2009 to 2015. Fig. S8: Proportion of all elective inpatient HES admissions with dementia, depression, or diabetes for the 65+ from 2009 to 2015. Fig. S9: Proportion of all emergency inpatient HES admissions with dementia, depression, or diabetes for the 65+ from 2009 to 2015.

## Data Availability

The pseudonymised patient data that were used for this study can be accessed by contacting NHS Digital (see https://digital.nhs.uk/services/data-access-request-service-dars). Access to these data is subject to a data sharing agreement (DSA) containing detailed terms and conditions of use following protocol approval from NHS Digital. Documents such as the study protocol are not available.

## Abbreviations

NHS: National Health Service
HES: Hospital Episode Statistics
ICD-10: International Classification of Diseases, 10th revision
18-44s: People aged between 18 and 44 years
45-64s: People aged between 45 and 64 years
65 +: People aged 65 years and over
SD: Standard deviation
OR: Odds ratio
NIHR: National Institute of Health Research
