# Supplementary material for: Has multimorbidity and frailty in adult hospital admissions changed over the last 15 years? A retrospective study of 107 million admissions in England
Source: BMC Med. 2024 Sep 11;22:369. doi: 10.1186/s12916-024-03572-z (PMC11389502; doi:10.1186/s12916-024-03572-z)

**Additional File 1**

Table of Contents

[Figure S1: Age vs log(odds) of three or more comorbidities for acute admissions across England from 2006 to 2021 2](#_Toc174246160)

[Figure S2: Age vs log(odds) of three or more comorbidities for elective admissions across England from 2006 to 2021 2](#_Toc174246161)

[Figure S3: Age vs log(odds) of two or more frailty syndromes for acute admissions across England from 2006 to 2021 3](#_Toc174246162)

[Figure S4: Age vs log(odds) of two or more frailty syndromes for elective admissions across England from 2006 to 2021 3](#_Toc174246163)

[Table S1: List of conditions used to measure multimorbidity and their point prevalence at the beginning and end of the study 4](#_Toc174246164)

[Table S2: Percentages for the proportion of admission trends with 0 to 3+ conditions by age and admission type for 2006/7 and 2020/2021 4](#_Toc174246165)

[Table S3: List of syndromes used to measure frailty and their point prevalence at the beginning and end of the study 5](#_Toc174246166)

[Table S4: Percentages for the proportion of admission trends with 0 to 2+ frailty syndromes by age and admission type for 2006/7 and 2020/2021 5](#_Toc174246167)

[Figure S5: Proportion of all inpatient HES admissions with dementia, depression or diabetes aged 18-44 years from 2009 to 2015. 6](#_Toc174246168)

[Figure S6: Proportion of all inpatient HES admissions with dementia, depression, or diabetes aged 45-64 years from 2009 to 2015. 6](#_Toc174246169)

[Figure S7: Proportion of all inpatient HES admissions with dementia, depression, or diabetes aged 65+ from 2009 to 2015. 6](#_Toc174246170)

[Figure S8: Proportion of all elective inpatient HES admissions with dementia, depression, or diabetes for the 65+ from 2009 to 2015. 7](#_Toc174246171)

[Figure S9: Proportion of all emergency inpatient HES admissions with dementia, depression, or diabetes for the 65+ from 2009 to 2015. 7](#_Toc174246172)

# Figure S1: Age vs log(odds) of three or more comorbidities for acute admissions across England from 2006 to 2021


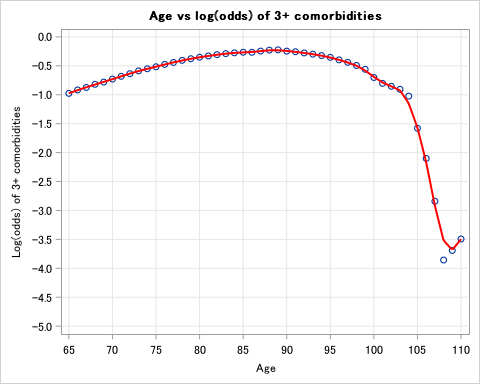


Spline selection: Spline knot applied at ages 90 and 103.

# Figure S2: Age vs log(odds) of three or more comorbidities for elective admissions across England from 2006 to 2021


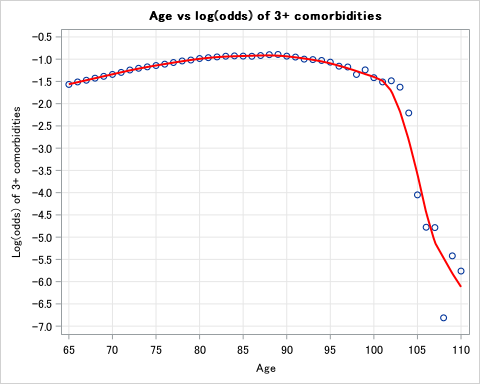


Spline selection: Spline knot applied at ages 90 and 103.

Frailty

# Figure S3: Age vs log(odds) of two or more frailty syndromes for acute admissions across England from 2006 to 2021


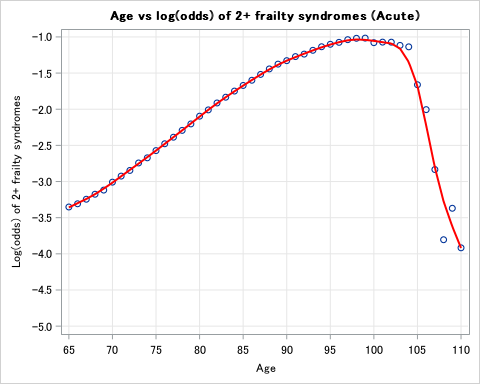


Spline selection: Spline knot applied at age 100.

# Figure S4: Age vs log(odds) of two or more frailty syndromes for elective admissions across England from 2006 to 2021


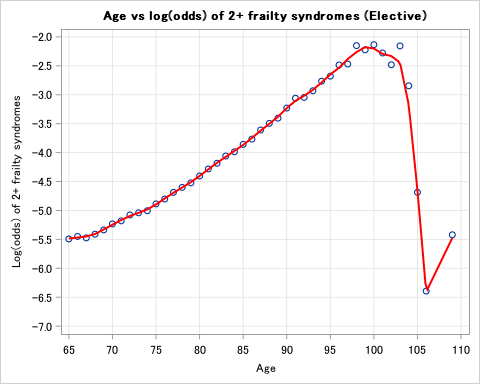


Spline selection: Spline knot applied at age 100.

# Table S1: List of conditions used to measure multimorbidity and their point prevalence at the beginning and end of the study

| **Condition** | **2006/2007 (%)** | **2020/2021 (%)** |
| --- | --- | --- |
| CHF | 3.0 | 7.5 |
| Arrhythmias | 7.3 | 15.4 |
| Valvular disease | 1.4 | 4.7 |
| Disease of pulmonary circulation | 0.5 | 1.6 |
| Peripheral vascular diseases | 1.8 | 3.6 |
| Hypertension (Combined HT with and without complications) | 16.4 | 30.4 |
| Paralytic syndromes | 0.7 | 1.2 |
| Other neurological diseases | 3.5 | 5.8 |
| Chronic pulmonary diseases | 9.6 | 18.8 |
| Diabetes (Combined diabetes with and without complications) | 8.1 | 15.2 |
| Thyroid disorders | 2.5 | 6.0 |
| Renal disease | 3.1 | 9.8 |
| Liver disease | 1.1 | 3.7 |
| Peptic ulcer | 0.2 | 0.3 |
| HIV* |  |  |
| Cancer (Combined Lymphoma, Metastasis and Solid tumour) | 7.8 | 9.5 |
| Connective tissue disorders | 1.5 | 3.5 |
| Coagulopathy | 0.3 | 1.1 |
| Obesity | 0.7 | 9.2 |
| Weight loss | 0.4 | 1.0 |
| Fluid disorders | 1.7 | 7.6 |
| Anaemia (Combined Blood loss anaemia and Deficiency anaemia) | 1.0 | 3.0 |
| Alcohol-related diseases | 3.2 | 5.0 |
| Substance abuse | 0.6 | 1.7 |
| Psychoses | 0.7 | 1.2 |
| Depression | 1.9 | 10.1 |
| Dementia^3^ (Charlson) | 1.9 | 5.0 |

* Note HIV was not included as it was omitted in our data extract due to sensitivity.

# Table S2: Percentages for the proportion of admission trends with 0 to 3+ conditions by age and admission type for 2006/7 and 2020/2021

|  |  | |  | | |  | | |  | | |  |  |  |  |
| --- | --- | --- | --- | --- | --- | --- | --- | --- | --- | --- | --- | --- | --- | --- | --- |
|  | |  | | |  | | |  | | |  | | |  |  |
| Acute | | 18-44 | | |  | | | 45-64 | | |  | | | 65+ |  |
| Number of comorbidities | | 2006/2007 | | | 2020/2021 | | | 2006/2007 | | | 2020/2021 | | | 2006/2007 | 2020/2021 |
| 0 | | 68.8 | | | 49.98 | | | 41.12 | | | 23.95 | | | 25.00 | 7.81 |
| 1 | | 22.57 | | | 27.12 | | | 32.95 | | | 25.91 | | | 31.35 | 15.91 |
| 2 | | 6.47 | | | 13.43 | | | 16.55 | | | 21.00 | | | 23.83 | 21.26 |
| 3+ | | 2.15 | | | 9.47 | | | 9.39 | | | 29.13 | | | 19.83 | 55.02 |
|  | |  | | |  | | |  | | |  | | |  |  |
| Elective | | 18-44 | | | | | | 45-64 | | | | | | 65+ | |
| Number of comorbidities | | 2006/2007 | | | 2020/2021 | | | 2006/2017 | | | 2020/2021 | | | 2006/2017 | 2020/2021 |
| 0 | | 89.79 | | | 63.66 | | | 48.96 | | | 24.77 | | | 34.58 | 14.11 |
| 1 | | 8.41 | | | 26.71 | | | 32.93 | | | 31.46 | | | 34.90 | 24.18 |
| 2 | | 1.38 | | | 7.47 | | | 12.19 | | | 21.61 | | | 18.69 | 24.22 |
| 3+ | | 0.42 | | | 2.16 | | | 5.92 | | | 22.16 | | | 11.84 | 37.49 |
|  |  | | |  | | |  | | |  | | |  |  |  |
|  |  | | |  | | |  | | |  | | |  |  |  |

# Table S3: List of syndromes used to measure frailty and their point prevalence at the beginning and end of the study

| **Frailty syndrome** | **2006/07 Prevalence** | **2020/2021 Prevalence** |
| --- | --- | --- |
| Dementia | 2.9 | 6.8 |
| Delirium | 0.1 | 2.4 |
| Mobility Problems | 0.7 | 3.8 |
| Falls and Fractures | 6.5 | 8.2 |
| Pressure Ulcers | 0.3 | 1.7 |
| Incontinence | 0.4 | 1.6 |
| Dependence and Care | 0.4 | 0.3 |
| Anxiety and Depression | 2.2 | 14.3 |
| Senility | 0.9 | 2.8 |

| Table S4: Percentages for the proportion of admission trends with 0 to 2+ frailty syndromes by age and admission type for 2006/7 and 2020/2021 | | | | | | |
| --- | --- | --- | --- | --- | --- | --- |
| Acute | 18-44 | | 45-64 | | 65+ | |
| Number of frailty syndromes | 2006/07 | 2020/21 | 2006/07 | 2020/21 | 2006/07 | 2020/21 |
| 0 | 89.45 | 74.86 | 87.12 | 70.93 | 72.62 | 53.4 |
| 1 | 10.27 | 23.43 | 12.14 | 24.75 | 21.14 | 28.19 |
| 2+ | 0.28 | 1.72 | 0.73 | 4.32 | 6.25 | 18.41 |
|  |  |  |  |  |  |  |
| Elective | 18-44 | | 45-64 | | 65+ | |
| Number of frailty syndromes | 2006/07 | 2020/21 | 2006/07 | 2020/21 | 2006/07 | 2020/21 |
| 0 | 98.33 | 85.78 | 96.78 | 82.90 | 94.67 | 85.84 |
| 1 | 1.64 | 14.03 | 3.07 | 16.14 | 4.56 | 12.38 |
| 2+ | 0.03 | 0.19 | 0.15 | 0.96 | 0.77 | 1.78 |

Sensitivity analysis - HES Coding Review for all admissions

# Figure S5: Proportion of all inpatient HES admissions with dementia, depression or diabetes aged 18-44 years from 2009 to 2015.


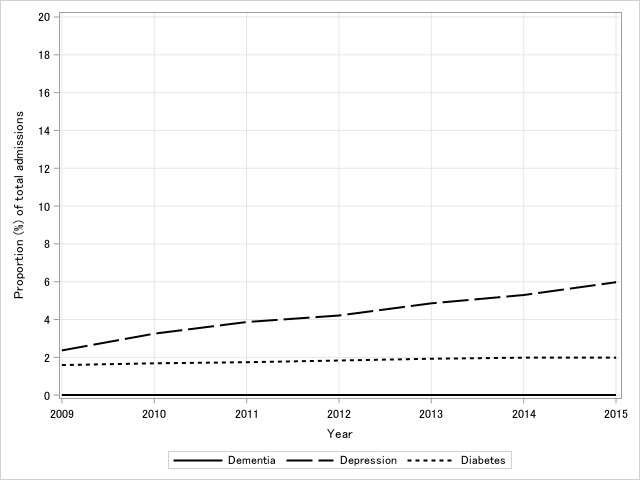


# Figure S6: Proportion of all inpatient HES admissions with dementia, depression, or diabetes aged 45-64 years from 2009 to 2015.


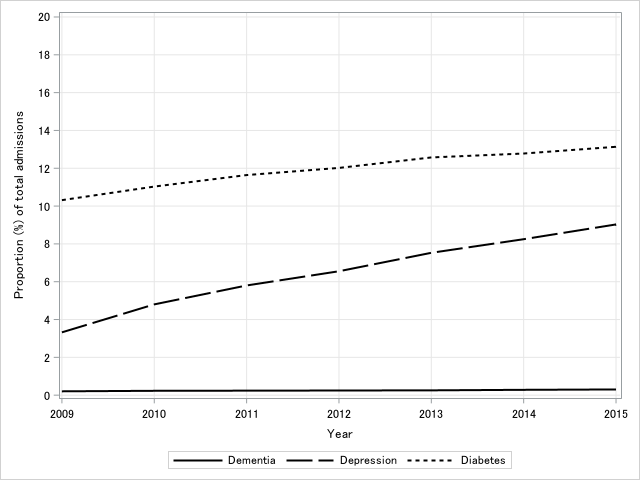


# Figure S7: Proportion of all inpatient HES admissions with dementia, depression, or diabetes aged 65+ from 2009 to 2015.


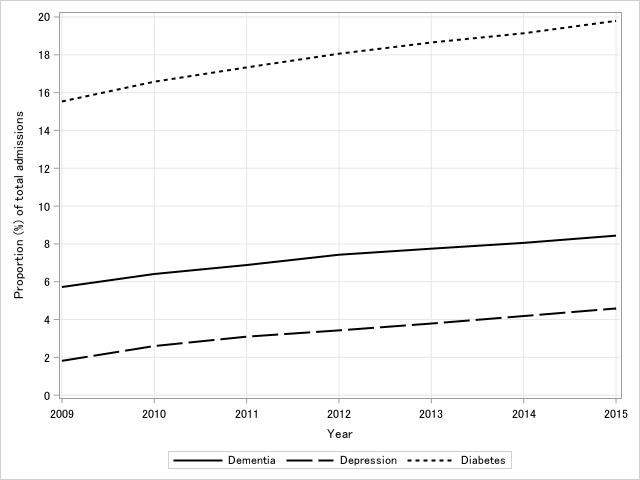


# Figure S8: Proportion of all elective inpatient HES admissions with dementia, depression, or diabetes for the 65+ from 2009 to 2015.


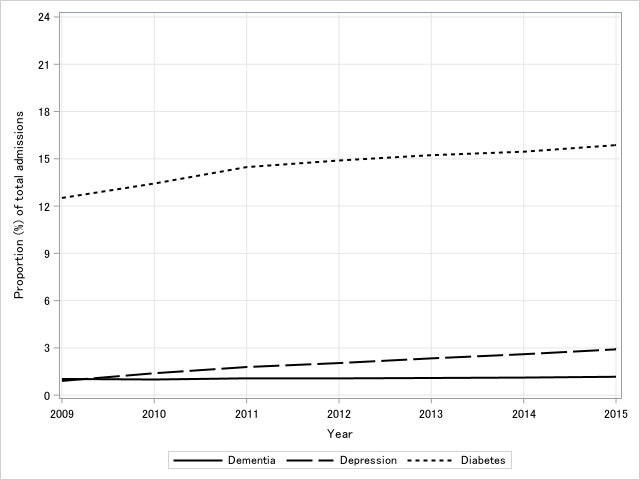


# Figure S9: Proportion of all emergency inpatient HES admissions with dementia, depression, or diabetes for the 65+ from 2009 to 2015.


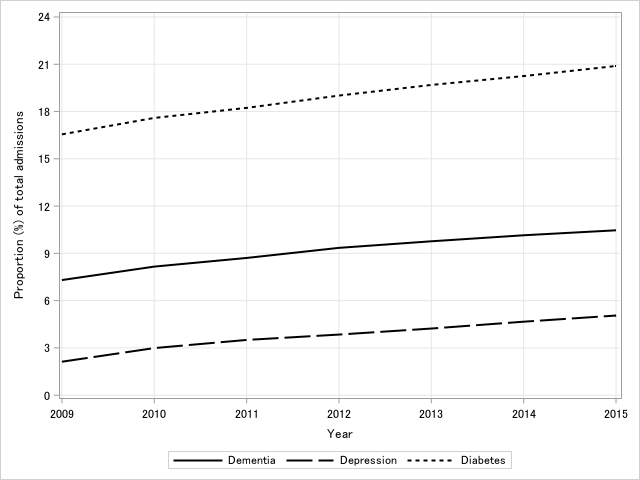

Supplement: Supplementary file 1 — Additional File 1: Fig. S1: Age vs log (odds) of three or more comorbidities for acute admissions across England from 2006 to 2021. Fig. S2: Age vs log (odds) of three or more comorbidities for elective admissions across England from 2006 to 2021. Fig. S3: Age vs log (odds) of two or more frailty syndromes for acute admissions across England from 2006 to 2021. Fig. S4: Age vs log (odds) of two or more frailty syndromes for elective admissions across England from 2006 to 2021. Table S1: List of conditions used to measure multimorbidity and their point prevalence at the beginning and end of the study. Table S2: Percentages for the proportion of admission trends with 0 to 3+ conditions by age and admission type for 2006/7 and 2020/2021. Table S3: List of syndromes used to measure frailty and their point prevalence at the beginning and end of the study. Table S4: Percentages for the proportion of admission trends with 0 to 2+ frailty syndromes by age and admission type for 2006/7 and 2020/2021. Fig. S5: Proportion of all inpatient HES admissions with dementia, depression or diabetes aged 18-44 years from 2009 to 2015. Fig. S6: Proportion of all inpatient HES admissions with dementia, depression, or diabetes aged 45-64 years from 2009 to 2015. Fig. S7: Proportion of all inpatient HES admissions with dementia, depression, or diabetes aged 65+ from 2009 to 2015. Fig. S8: Proportion of all elective inpatient HES admissions with dementia, depression, or diabetes for the 65+ from 2009 to 2015. Fig. S9: Proportion of all emergency inpatient HES admissions with dementia, depression, or diabetes for the 65+ from 2009 to 2015. [file 12916_2024_3572_MOESM1_ESM.docx]
